# Simulation of Human Identification Using DNA From Formalin-Fixed and Paraffin-Embedded (FFPE) Cancer Tissue as a Reference Sample

**DOI:** 10.7759/cureus.96712

**Published:** 2025-11-12

**Authors:** Marta Ortega-Martínez, María-de-Lourdes Chávez-Briones, Mariana Moreno-Mares, Ivett Miranda-Maldonado, Alyna Galindo-Gómez, Yareth Gopar-Cuevas, Adriana Ancer-Arellano, Gilberto Jaramillo-Rangel

**Affiliations:** 1 Department of Pathology, School of Medicine, Autonomous University of Nuevo León, Monterrey, MEX

**Keywords:** cancer, dna analysis, forensic science, formalin-fixed and paraffin-embedded (ffpe) tissues, human remains identification

## Abstract

Archived slides of formalin-fixed and paraffin-embedded (FFPE) tissues are often the only source of DNA available for the identification of a deceased person. Difficulties may arise when FFPE tissue from a malignant tumor is used. We performed a simulated identification of human remains to estimate how useful the use of such type of samples would be in a real-life scenario. Tissue samples obtained from a patient with clear cell ovarian carcinoma were processed according to standard histopathological procedures. Nine years later, three slides (representing reference samples of a missing person) were selected: one containing only healthy tissue, one with a mixture of healthy and cancerous tissue, and one with only cancerous tissue. A saliva swab (representing a sample of found human remains) was obtained from the patient. A short tandem repeat (STR) profile was obtained from the samples. Healthy tissue showed a complete loss of two alleles. The mixture of healthy and cancerous tissue showed a complete loss of three alleles. The complete genetic profile of the sample containing only cancerous tissue was obtained, which matched all alleles with the one obtained from saliva. None of the tissue samples showed the presence of additional alleles. The findings presented in this work indicate that, in some cases, FFPE samples containing cancerous tissue could be used as reference samples in the genetic identification of human remains. However, caution should be exercised when abnormalities are present that could complicate the identification process, such as a complete loss of alleles or the presence of additional alleles.

## Introduction

Short tandem repeat (STR) analysis is a standard genotyping method used in the field of forensic science. Archived slides of formalin-fixed and paraffin-embedded (FFPE) tissues are often the only source of DNA available for forensic investigations, including the identification of a deceased person, like in criminal cases, disaster victim identification, or medicolegal investigations where traditional reference samples are absent. Unfortunately, DNA extracted from FFPE tissues is frequently scarce and degraded, and contains traces of xylene, which inhibits the proteinase K used in the DNA extraction procedure, and formalin, which inhibits the amplification reaction [1,2]. Also, it has been reported that the storage time of the slides may influence the quality of the extracted DNA and result in samples not being correctly identified by STR genotyping [3].

In some specific cases of human remains identification, additional difficulties may arise, such as when only FFPE tissue from a malignant tumor is available as a reference sample for comparison with the sample in question. Tumor DNA could harbor genetic changes, such as loss of heterozygosity (LOH) and microsatellite instability (MSI), which, if concerning the STRs used in the identification process, may lead to misinterpretation of results [4-6]. Most studies in the area are focused on evaluating the incidence of allelic alterations in STRs used in forensic identification. For this purpose, these studies analyze whether specific STRs are mutated or remain in their normal state by comparing cancer tissues with reference tissues (or sometimes blood samples) derived from the same individuals. The allelic alterations investigated in these studies have been MSI, which is characterized by allelic insertion, and LOH, characterized by allelic deletion, with partial or complete loss of an allele [4,5,7-11]. Case report articles dealing with the identification of a person using FFPE tissue from a malignant tumor are very scarce in the literature. In those articles, gastric [12], lung [13], and colorectal [14] tumor tissues were analyzed.

Based on the above, controversy remains regarding the usefulness of FFPE cancer tissue samples for forensic identification. Therefore, we consider it important to continue investigating this issue. In this work, we present the results of a simulation in which a STR profile obtained from FFPE tissues used in 2016 for the diagnosis of a patient with clear cell ovarian carcinoma (representing the reference profile of a missing person) was compared with a STR profile from a saliva sample from the same patient obtained in 2025 (representing the profile of found human remains). This was the first time that clear cell ovarian carcinoma was analyzed in this forensic context, and that cancerous tissue, healthy tissue, and a mixture of both tissues from the same subject were studied, which allowed for more solid conclusions.

## Case presentation

In October 2016, a patient underwent a hysterectomy and bilateral salpingo-oophorectomy because her clinical signs and symptoms and laboratory test results suggested she had ovarian cancer. Histopathological analysis revealed a diagnosis of clear cell ovarian carcinoma. The patient subsequently underwent chemotherapy and received appropriate follow-up. She has not shown any signs of recurrence and is in good health to date.

Tissue samples obtained from the patient were fixed in formalin, embedded in paraffin, cut into 5-µm-thick sections, mounted on slides, and stained with hematoxylin and eosin according to standard histopathological procedures. After being analyzed to obtain the diagnosis, the slides were stored in the dark at room temperature.

**Figure 1:**
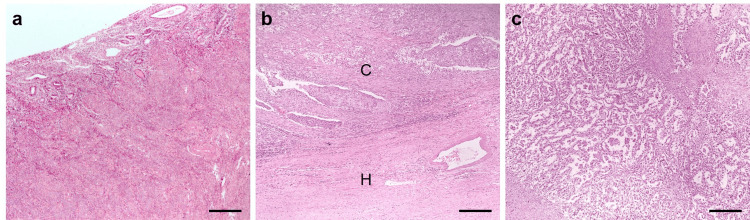
Representative microphotographs of the tissues analyzed (a) Uterus section containing only healthy tissue; (b) ovary section containing a mixture of healthy (H) and cancerous (C) tissue; (c) ovary section containing only cancerous tissue. Bar = 100 µm.

Nine years later (in 2025), three slides were selected (Figure 1): one containing only healthy tissue (uterus), one with a mixture of healthy and cancerous tissue (ovary), and finally one with only cancerous tissue (ovary).

The coverslips were removed from the slides by soaking in xylene at 56°C. Tissue sections were dewaxed by three successive immersions in xylene for 5 minutes each. The sections were subsequently rehydrated using a descending alcohol series (100%, 2 x 5 minutes; 95%, 3 minutes; 70%, 3 minutes). Hematoxylin and eosin staining was then removed by washing the samples in 70% ethanol containing 1% HCl for 3 minutes. Finally, tissues were excised with sterile surgical blades and transferred into sterile microtubes containing 1 mL distilled water.

DNA extraction was performed using the PrepFiler Express BTA™ Forensic DNA Extraction Kit (Applied Biosystems, Foster City, CA, USA). For this purpose, lysis buffer from the kit was added to the tissues together with proteinase K (200 μg/mL) and 1 M DTT. Samples were incubated overnight at 56°C with gentle shaking. Finally, they were centrifuged, and the supernatant was subjected to DNA extraction in the AutoMate™ Instrument (Applied Biosystems) in accordance with the manufacturer’s instructions.

In addition, a saliva sample was obtained from the patient, and DNA was extracted from it using a Chelex protocol [15].

DNA samples were quantified using the Quantifiler™ HP DNA quantification kit on the 7500 ABI Real-Time PCR platform (Applied Biosystems). Simultaneously, the level of DNA degradation, expressed as degradation index (DI), was assessed for each sample. The DI value is a ratio of the concentration of a small amplicon (80 bp) compared to a long amplicon (214 bp). DNA was eluted in 30 µL of the kit's elution buffer. The DNA concentration and the DI obtained from the analyzed samples are summarized in Table 1.

**Table 1:** Yield and degradation index of DNA obtained from healthy tissue, a mixture of healthy and cancerous tissue, and only cancerous tissue from the same patient

Sample	DNA concentration (ng/µL)	Degradation index (DI)
​Healthy	1.5	​ 31
​Healthy/cancerous	2.3	​ 23
​Cancerous	8.7	​ 17

DNA samples (1 ng) were amplified using the GlobalFiler™ PCR Amplification Kit (Applied Biosystems) following the protocol provided by the manufacturer. Alleles were separated and detected with ABI PRISM® 3500 genetic analyzer and GeneMapper® IDX-v1.6 software (Applied Biosystems). A detection limit threshold of 55 relative fluorescence units (RFU) was used for allele labeling, and a stochastic threshold of 300 RFU was set for designation of homozygotes. Polymerase chain reaction (PCR) amplification and electrophoresis were repeated three times for all samples to verify the reproducibility of allele calling. LOH was calculated using the following equation: $\text{LOH} = \dfrac{(\text{height of normal allele 2} / \text{height of normal allele 1})}{(\text{height of tumor allele 2} / \text{height of tumor allele 1})}$.

The reasoning applied in the formula consists of measuring the deviation of the allelic proportion between normal and cancerous tissue, normalizing and quantifying that deviation through a ratio relationship. LOH occurs when the resulting value is less than 0.5 or greater than 2.0 [4,6].

Healthy tissue showed complete loss of both alleles at locus TPOX, and LOH at loci D18S51, FGA, and D12S391. The mixture of healthy and cancerous tissue showed complete loss of both alleles at locus TPOX, the complete loss of an allele at locus SE33, and LOH at loci D2S441, FGA, and D12S391. Finally, the sample that contained only cancerous tissue showed LOH at loci D2S441, FGA, and D7S820. None of the tissue samples showed the presence of additional alleles (Tables 2, 3).

**Table 2:** Comparison of short tandem repeats results of DNA recovered from saliva, healthy tissue, a mixture of healthy and cancerous tissue, and only cancerous tissue from the same patient ^a^ No results were obtained; ^b^ Allele 27.2 was not obtained.

Locus	Saliva	Healthy	Healthy/Cancerous	Cancerous
​D3S1358	15-15	15-15	​ 15-15	​15-15
​vWA	16-16	16-16	​ 16-16	​16-16
​D16S539	11-11	11-11	​ 11-11	​11-11
​CSF1P0	10-11	10-11	​ 10-11	​10-11
​TPOX	8-8	NR^a^	​NR^a^	​8-8
​D8S1179	13-14	13-14	​ 13-14	​13-14
​D21S11	30-32.2	30-32.2	​ 30-32.2	​30-32.2
​D18S51	11-18	11-18	​ 11-18	​11-18
​D2S441	12-14	12-14	​ 12-14	​12-14
​D19S433	13-13	13-13	​ 13-13	​13-13
​TH01	6-9	6-9	​ 6-9	​6-9
​FGA	22-25	22-25	​ 22-25	​22-25
​D22S1045	15-16	15-16	​ 15-16	​15-16
​D5S818	11-12	11-12	​ 11-12	​11-12
​D13S317	8-9	8-9	​ 8-9	​8-9
​D7S820	10-11	10-11	​ 10-11	​10-11
​SE33	17-27.2	17-27.2	​ 17^b^	​17-27.2
​D10S1248	12-13	12-13	​ 12-13	​12-13
​D1S1656	15-17.3	15-17.3	​ 15-17.3	​15-17.3
​D12S391	17-23	17-23	​ 17-23	​17-23
​D2S1338	20-20	20-20	​ 20-20	​20-20
​Amelogenin	XX	XX	​ XX	​XX

**Table 3:** LOH values obtained from healthy tissue, a mixture of healthy and cancerous tissue, and only cancerous tissue from the same patient ^a^ Not applicable; ^b^ Loci with LOH are marked in bold. LOH: loss of heterozygosity

Locus	Healthy	Healthy/Cancerous	Cancerous
​D3S1358	-^a^	​ -	​-
​vWA	-	​ -	​-
​D16S539	-	​ -	-
​CSF1P0	0.84	​ 0.54	​0.69
​TPOX	-	​-	​-
​D8S1179	0.98	​ 0.84	​0.80
​D21S11	1.18	​ 1.25	​1.11
​D18S51	2.41^b^	​ 1.40	​1.20
​D2S441	1.26	​ 2.35	​2.25
​D19S433	-	​ -	​-
​TH01	1.58	​ 0.72	​1.51
​FGA	2.20	​ 5.08	​4.87
​D22S1045	1.08	​ 1.02	​1.00
​D5S818	1.02	​ 0.65	​0.74
​D13S317	1.23	​ 0.78	​0.67
​D7S820	1.20	​ 0.92	​3.10
​SE33	1.05	​ -	​0.92
​D10S1248	1.26	​ 1.20	​0.66
​D1S1656	0.90	​ 1.25	​1.21
​D12S391	4.23	​ 2.17	​0.90
​D2S1338	-	​ -	​-
​Amelogenin	-	​ -	​-

**Figure 2:**
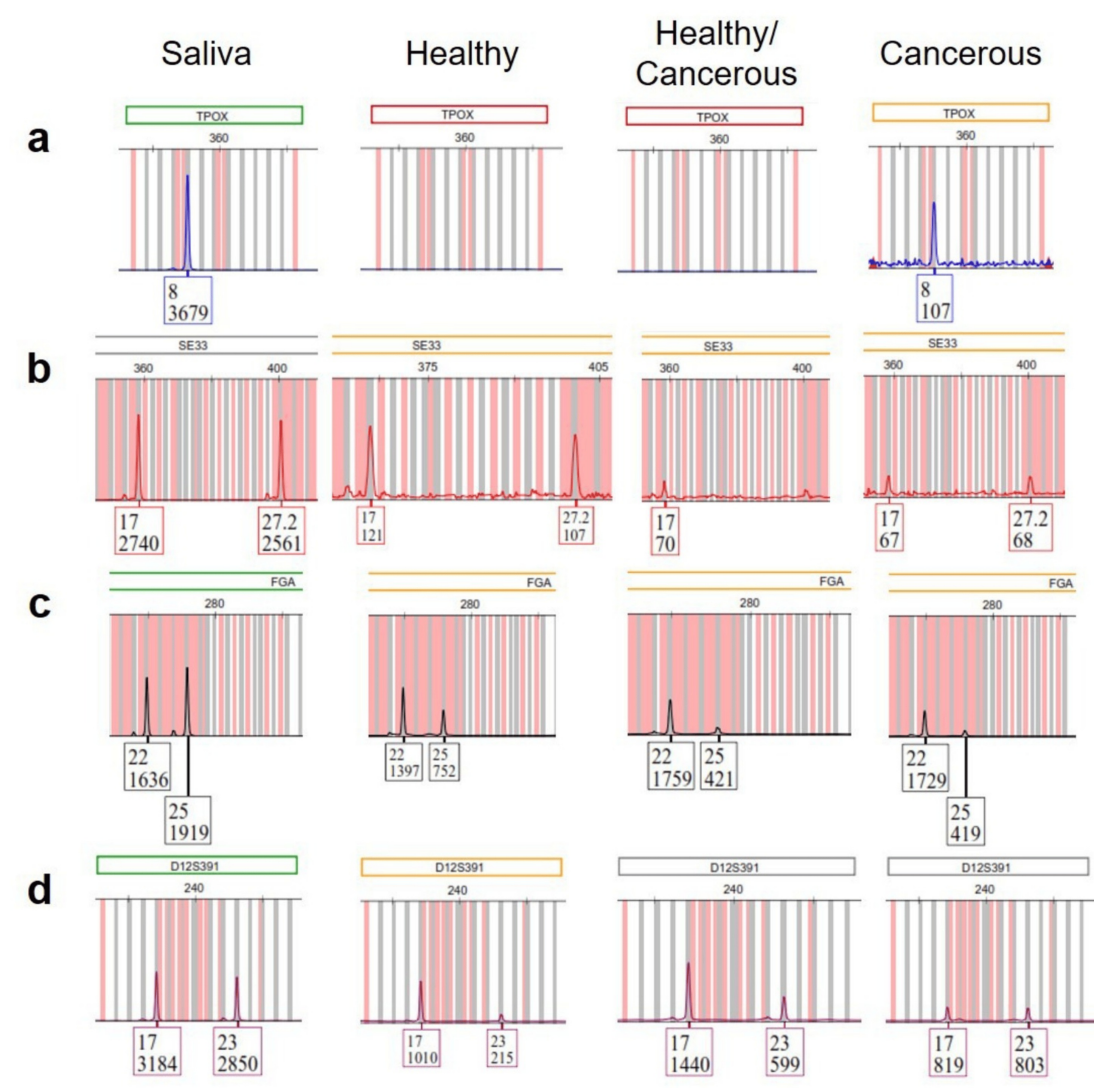
Fragments of electropherograms showing examples of alterations found in the study (a) The healthy tissue and the mixture of healthy and cancerous tissue had complete loss of both alleles at locus TPOX; (b) the mixture of healthy and cancerous tissue showed the complete loss of an allele at locus SE33; (c) all tissue samples had LOH at FGA locus; (d) the healthy tissue and the mixture of healthy and cancerous tissue showed LOH at locus D12S391. LOH: loss of heterozygosity

Figure 2 shows fragments of electropherograms with examples of alterations described above.

The random match probability was calculated using STR allele frequency data from our population and PATPCR software version 2.0.2 (National Institute of Forensic Science, Canberra, Australia) [16,17]. The random match probability was 1 in 9.26 x 10^25^ for the healthy tissue, 1 in 1.17 x 10^24^ for the mixture of healthy and cancerous tissue, and finally 1 in 3.47 x 10^26^ for the sample that contained only cancerous tissue.

## Discussion

Archived FFPE tissue is sometimes the only source of antemortem material from which reference DNA profiles can be generated to identify human remains. However, this FFPE DNA can present problems in the identification process due to its degradation caused by various factors; in addition, nucleotide changes may be observed when FFPE tissue from a malignant tumor is processed. In this study, we performed a simulated identification of human remains to estimate how useful the use of such type of samples would be in a real-life scenario.

In clinical practice, the observation of LOH is an important prognostic factor when analyzing tumor behavior. However, LOH is of relative importance for forensic purposes and is unlikely to lead to incorrect genotyping. In the case presented here, the majority of the genetic alterations observed in the tissue samples were LOH (Table 3).

In contrast, the presence of additional alleles or the complete loss of alleles can introduce significant problems in the interpretation of results and lead to false exclusions in identification cases, especially when mutational phenomena occur at more than two loci [4,9]. In none of the samples was the presence of additional alleles observed. Complete allele loss was detected at one locus in the healthy tissue sample, and at two loci in the sample containing the mixture of healthy and cancerous tissue. The complete genetic profile of the sample containing only cancerous tissue was obtained, which matched all alleles with the one obtained from saliva (Table 2). Thus, considering the above and the values ​​of random match probability obtained, it is evident that the comparison of the genetic profile obtained from any of the tissue samples with that from saliva would have led to the identification of the supposed human remains. Although the random match probability threshold that confers certainty that a genetic profile is unique in our population has not been calculated, this value is in the order of 1 in 1 x 10^9^-1 in 1 x 10^10^ in other populations [18,19]. The random match probability values ​​observed in the samples processed in this study were between 1 in 1.17 x 10^24^ and 1 in 3.47 x 10^26^. The identification kit used in this work (GlobalFiler) has been validated in our population. The combined power of discrimination was >0.99999999999999, and the random match probability range was 1 in 1.23 to 3.0 x 10^25^ [20]. Therefore, the results obtained in this study can be considered reliable.

It is important to note that this study involved a direct comparison between reference and questioned samples, and that the mutational phenomena found could be more significant and cause a greater number of problems if the comparison had been between relatives, for example, in a paternity test. However, cases of paternity assignment using mutated DNA from cancerous tissue as a reference sample in the parentage test have been reported. Canan and Serin [13] compared the STR profiles of an individual and his suspected father. Since the alleged father was deceased, FFPE samples obtained from him containing lung cancer tissue were analyzed. As a result, mismatches were found at two loci, and one locus of the presumed father presented three alleles. Even so, the final probability of paternity value was higher than 99.999%.

In another paternity test, Liu et al. [12] compared a blood sample from one individual and FFPE samples containing cancerous gastric tissue from the alleged father. As a strategy to obtain better results, the authors separately processed the cancerous and non-cancerous tissue fractions of FFPE samples. The STR profile of non-cancerous tissue showed no allelic alterations, whereas cancerous tissue showed the complete loss of an allele at one locus and the presence of an additional allele at another locus. The authors did not report the paternity probability value that would have been obtained by considering the genetic profile from the cancerous tissue.

The two paternity tests described above correspond to two of the three case reports mentioned in the Introduction. In the third, Soldati et al. [14] analyzed two FFPE samples from the same subject, one with colorectal cancer tissue and the other with normal tissue, using three autosomal STR amplification kits. Compared with normal tissue, three genomic instability phenomena in the cancer tissue were found: the complete loss of an allele at two loci and the addition of an allele at another locus. Since even FFPE samples of normal tissue can produce altered genetic profiles, it would have been interesting in this case to compare the results obtained with those from samples such as blood or saliva from the same individual to determine the usefulness of both FFPE samples in forensic identification.

Due to the specific characteristics of each forensic identification case involving FFPE samples of cancerous tissue (e.g., whether it is a direct identification or a paternity test, the specific type of cancer, the availability of non-FFPE samples such as blood or saliva), it is difficult to establish a single methodological strategy applicable to all situations. Since FFPE samples of cancerous tissue have the potential to exhibit genetic instability, it would be logical to avoid their use in the identification process. However, our findings and those of other authors (in addition to the case report described above [13]) indicate that such samples should not necessarily be excluded in forensic identification cases. Budimlija et al. [5] compared FFPE tissues from 13 different types of malignant tumors with healthy tissues from the same individuals. The percentage of complete genetic profiles obtained was similar between both types of samples (44% vs. 41%, for tumor and healthy samples, respectively). Poetsch et al. [4] analyzed FFPE samples from 118 primary tumor lesions and 62 metastases from four solid tumor types. They found events that would lead to a wrong STR typing result, i.e., the complete loss of alleles or the presence of additional alleles, in only 20% of the cancer samples analyzed. Therefore, it is advisable not to rule out a priori the analysis of FFPE samples containing cancerous tissue.

Interestingly, in this work, no allele loss was observed in the sample containing only cancerous tissue, while such an alteration was found in the other two samples. In addition to the type of tissue analyzed, there are multiple processing factors that can influence the results of the DNA analysis of FFPE samples, including specimen size, fixative used (neutral buffered formalin or unbuffered formalin), temperature and duration of fixation, section thickness, and storage time of the tissue sections [21,22]. Although we do not know the exact treatment to which each sample was subjected, it is likely that all three were processed under similar conditions and on similar dates. Thus, the variability in the results obtained could be attributed only to the type of tissue analyzed. However, variability has been observed in the results obtained from the analysis of similar FFPE samples even when they were processed in the same pathology department and on similar dates [23,24]. Further research is needed to determine the causes of such variability and the significance it may have on the results of forensic identification cases.

Results of genetic analyses are dependent on the quantity and quality of DNA extracted from the biological samples. The DNA concentration obtained from the sample containing only cancerous tissue was higher, and the DI was lower than those obtained from the other two samples (Table 1). However, the amount of DNA analyzed by PCR was the same for all three samples (1 ng). Therefore, the allele loss observed in the sample containing only healthy tissue and the one containing a mixture of healthy and cancerous tissue could be due to greater fragmentation of their DNA molecules (reflected in higher DIs), resulting in shorter target sequences for PCR. Indeed, the missing alleles (TPOX and SE33) are the two with the largest amplicons in the GlobalFiler kit [25,26].

Finally, it is important to clarify that we do not intend to assert that, in forensic identification processes, it is better to use samples containing cancerous material as a reference rather than samples of healthy tissue. In fact, in this case, all three samples were highly informative, and any could be used in an identification process. Furthermore, as discussed in the previous paragraphs, differences between the samples could be due to factors unrelated to the nature of the tissues themselves. Analyzing a larger number of identification cases and conducting case-control studies to determine the incidence of alterations in STRs of forensic interest, specifically in clear cell ovarian carcinoma, would contribute to a better understanding of the findings observed in this study. It would also be interesting to analyze FFPE samples of cancer tissue with other tests, such as insertion/deletion (InDel) polymorphisms, single-nucleotide polymorphisms (SNPs), and next-generation sequencing, to find out what genetic alterations might be present in this type of sample and whether they alter the forensic identification process.

## Conclusions

The findings presented in this work indicate that, in some cases, FFPE samples containing cancerous tissue could be used as reference samples in the identification of human remains by means of STR typing. However, caution should be exercised when abnormalities are present that could complicate the identification process, such as MSI, which is characterized by allelic insertion, and LOH, characterized by allelic deletion, with partial or complete loss of an allele. Because this is a case report study, more investigation is required to draw more generalizable conclusions.
